# The usefulness of intraoperative electrocorticography (iECoG) in pediatric temporal lobe epilepsy surgery

**DOI:** 10.1002/epd2.70160

**Published:** 2026-01-12

**Authors:** Rafael Andrade Cruz, João Paulo Sant’ Ana Santos de Souza, Davi Casale Aragon, Úrsula Thomé Costa, Ana Paula Andrade Hamad, Américo Ceiki Sakamoto, Antônio Carlos dos Santos, Hélio Rubens Machado, Marcelo Volpon Santos

**Affiliations:** ^1^ Center of Epilepsy Surgery (CIREP), Pediatric Section, Ribeirão Preto Medical School University of São Paulo São Paulo Brazil; ^2^ Statistician, Department of Pediatrics, Ribeirão Preto Medical School University of São Paulo São Paulo Brazil; ^3^ Division of Neurosurgery, Department of Surgery and Anatomy, Ribeirão Preto Medical School University of São Paulo São Paulo Brazil; ^4^ Division of Neuroradiology, Department of Medical Imaging, Hematology and Clinical Oncology, Ribeirão Preto Medical School University of São Paulo São Paulo Brazil

**Keywords:** intraoperative electrocorticography, mesial temporal lobe sclerosis, temporal lobe epilepsy

## Abstract

**Objective:**

This study aimed to evaluate the usefulness of intraoperative electrocorticography (iECoG) in providing a more accurate surgical strategy, thereby yielding seizure freedom following resective surgery in children with temporal lobe epilepsy (TLE).

**Methods:**

The authors conducted a retrospective review of pediatric patients with drug‐resistant TLE due to various etiologies, within a relatively long follow‐up (range 8.5–11.5 years). Patients were divided into two groups based on whether they were operated on using iECoG or not, which was employed in cases of uncertain delineation of the EZ or anticipated extended resection. The efficacy of surgical treatment was assessed using Engel's classification. Seizure‐freedom rate for each etiology was compared between groups using Fisher's exact test with a 95% confidence interval.

**Results:**

A total of 81 patients were included in the study (mean age 11.8 years, range 1–18 years), of whom 63 (77.8%) achieved Engel I status after 10 years. The main etiology was hippocampal sclerosis (34/81, 41.9%), followed by tumors (25/81, 30.8%) and focal cortical dysplasia (22/81, 27.1%). iECoG was performed in 29 (35.8%) patients. Overall, there were no significant differences in the proportion of Engel I (*p* = .78) among those who performed iECoG (22/29, 75.9%) and did not perform iECoG (*n* = 41/52, 78.8%). Among tumor‐associated cases, Engel I was achieved in 100% of patients with iECoG, compared with 76.5% without iECoG (*p* = .27). No significant differences were observed in focal cortical dysplasia (*p* = .61) or hippocampal sclerosis (*p* = .35).

**Significance:**

The study did not show that intraoperative iECoG improved Engel class I outcomes. Refinement of iECoG methods and future studies controlling for confounders are warranted.

**Key points**

- Intraoperative electrocorticography (iECoG) was utilized in 35.8% of pediatric temporal lobe epilepsy surgeries.
- Long‐term seizure freedom (Engel Class I) was achieved in 77.8% of patients after a mean follow‐up of 10 years.
- The use of iECoG did not significantly enhance seizure freedom rates across different etiological groups.
- A potential selective advantage of iECoG was observed in tumor‐associated epilepsy.
- Prospective, etiology‐specific investigations are warranted to further elucidate its clinical utility.

## 1 INTRODUCTION

Resective surgery has become established as the main treatment for drug‐resistant temporal lobe epilepsy (TLE) in children, accounting for 23% of all pediatric epilepsy surgeries.^1^, ^2^ The rate of postoperative seizure freedom in children with TLE ranges from 60% to 86%.^1^ The extent of resection of the epileptogenic zone (EZ) is the main factor that influences surgical effectiveness and explains the heterogeneous seizure freedom rates among series.^3^

Delineating a clear anatomo‐electro‐clinical (AEC) hypothesis is of paramount importance for surgical planning and depends on the efforts of a multidisciplinary team.^4^, ^5^ Non‐invasive tools, including detailed analysis of seizure semiology, video‐electroencephalography (EEG), magnetoencephalography (MEG), and high‐resolution magnetic resonance imaging (MRI), are essential for delineating the AEC hypothesis that guides surgical planning. Additional neuroimaging techniques, such as functional MRI, ictal single photon emission computed tomography (SPECT), and positron emission tomography (PET), are also commonly employed.^6^

Invasive methods include intraoperative electrocorticography (iECoG), as well as chronic monitoring techniques such as subdural grids and stereoelectroencephalography (SEEG). These approaches are indicated when there is discordance between electroclinical features and MRI,^2^ the putative EZ overlaps eloquent cortex,^3^ or MRI is negative.^5^, ^7^ They rely on the placement of electrodes directly on cortical and/or subcortical areas to confirm, deny, and/or complement the AEC hypothesis.

The use of iECoG was first reported in the 1930s by Penfield and Jasper to record brain activity during surgery, and became a standard tool in preoperative planning.^8^ iECoG refers to the use of strips and grids that gather neurophysiologic data directly over the cerebral cortex, aiming to identify regions of significant epileptogenicity. Another purpose of iECoG recordings is to monitor epileptiform discharges provoked by cortical stimulation, also known as post‐discharges.^9^

Albeit an invaluable tool, the outcomes attributable to the use of iECoG in pediatric cohorts are not clear. The purpose of this study is to investigate the usefulness of iECoG in children with drug‐resistant TLE, secondary to hippocampal sclerosis (HS), tumor, and focal cortical dysplasia (FCD).

## 2 MATERIALS AND METHODS

### 2.1 Patients

A retrospective study was conducted and analyzed consecutive pediatric patients with drug‐resistant TLE who underwent resective surgery between 1995 and 2020 at the Epilepsy Surgery Center of Ribeirão Preto Medical School, University of São Paulo. Inclusion criteria were as follows: refractoriness of seizure control with at least two antiseizure medications, age under 18 years, and multidisciplinary preoperative evaluation.

Anatomopathological results were evaluated and patients with etiologies other than HS, FCD, and tumor were excluded from the analysis. Pediatric patients with less than 10 years of follow‐up were also excluded. Patients were divided into two groups according to the use (or not) of iECoG.

The study protocol was approved by the ethics committee of our institution, protocol number 78330924.8.0000.5440.

### 2.2 Preoperative evaluation and surgical techniques

Before our multidisciplinary preoperative board, all patients underwent a standard non‐invasive protocol that included a detailed clinical history and neurological examination, scalp and video‐electroencephalography, high‐resolution 3 T Magnetic Resonance Imaging (MRI), ictal SPECT and PET, neuropsychological testing, and social assessment.

iECoG was selectively employed in patients in whom a clear AEC hypothesis could not be established based on non‐invasive evaluation, or in cases where a broader temporal resection was being considered. The decision was made as part of the multidisciplinary surgical planning process.

In our center, iECoG was also considered in selected cases of HS when presurgical investigations suggested a possible extension of epileptogenic activity beyond the mesial temporal structures. For instance, subtle FCD detected on MRI or metabolic abnormalities identified on PET to better delineate the resection margins intraoperatively.

**FIGURE 1:**
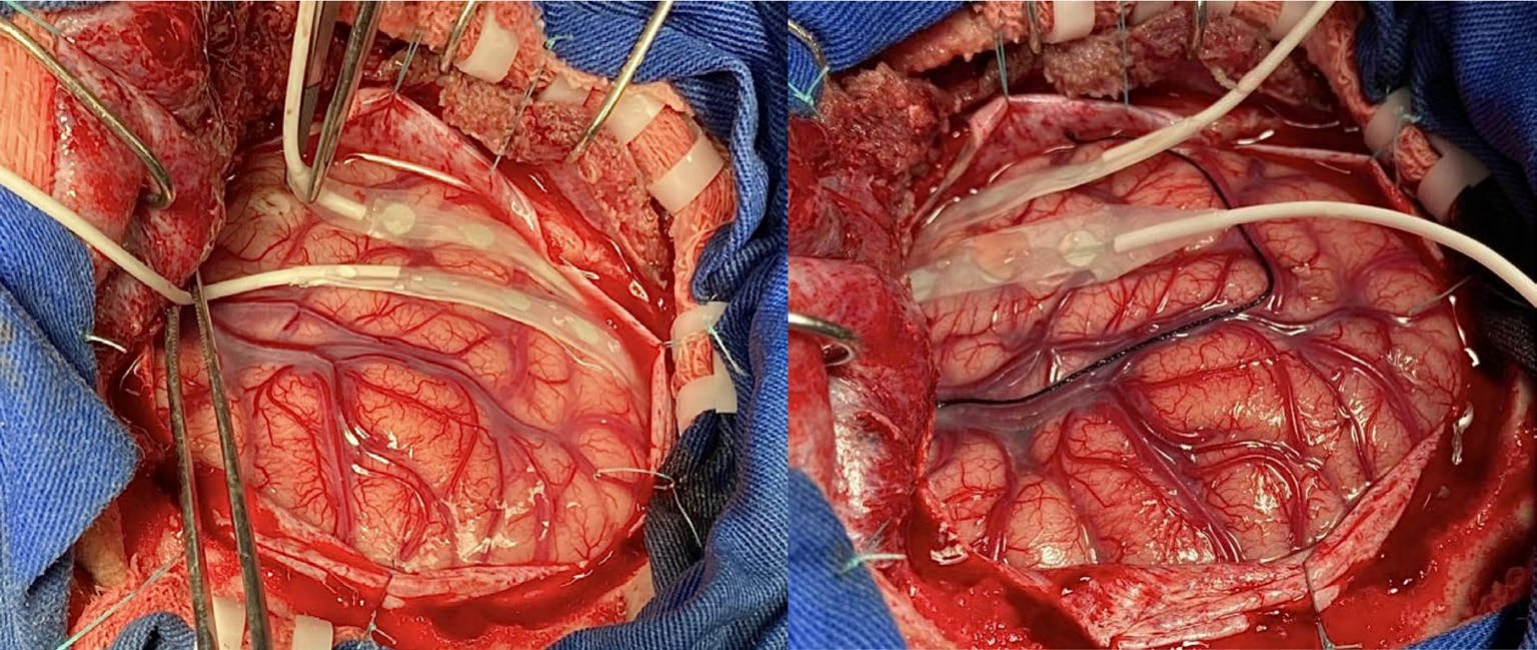
Example of placement of iECoG electrode strips over the temporal cortical surface during the surgical procedure.

In such cases, a larger craniotomy was designed for wider exposure, and 2–3 strips with 6 contacts each were placed upon the temporal cortex toward the polar, inferior, and posterior boundaries of the temporal lobe (Figure 1). Electrode strips with contact diameters of 4 mm and center‐to‐center spacing of 5 mm were used in all intraoperative recordings.

iECoG signals were recorded using a Nihon‐Kohden acquisition system, with a sampling rate of 500 Hz and band‐pass filters of 35–70 Hz. Intraoperative recordings were performed under general anesthesia following a preoperative discussion with the neurophysiology team. Neuromuscular blockade, as well as all anesthetic agents that could interfere with monitoring, were avoided.

The area to be resected was tailored according to the contacts, which showed epileptogenic abnormalities. Therefore, one of the following strategies would be pursued: (1) lesionectomy, when the EZ could be precisely delineated, especially if a localized lesion was seen on pre‐operative scans, and with preservation of the mesial structures; (2) lobectomy, which could be either standard, involving 4–5 cm of neocortical resection along with amygdalohypocampectomy, or extended, according to the intraoperative electrophysiological findings.

### 2.3 Statistical analysis

Clinical and demographic data were described as frequencies. Postoperative seizure‐freedom was evaluated according to Engel's classification. Seizure‐freedom rate was compared between those who underwent iECoG or not using Fisher's exact test with a confidence interval of 95%.

## 3 RESULTS

Over the study period, our service performed 140 operations for TLE on 135 pediatric patients, of which 81 were included herein. Among these patients, 63 (77.8%) achieved Engel I status after an average follow‐up of 10.0 years (range 8.5 to 11.5 years). The cohort included 41 males (50.6%) and 40 females (49.4%), with a mean age at surgery of 11.8 years (range 1 to 18 years). Overall, 29 (35.8%) of the surgical procedures involved intraoperative monitoring (Tables 1 and 2).

**TABLE 1:** Clinical and demographic characteristics of pediatric patients undergoing temporal lobe epilepsy surgery.

Characteristics	Value
Total	
Total of procedures	140
Total of patients	135
Patients included in the study	
Number of patients	81
Patients with Engel I status post‐surgery	63 (77.8%)
Utilization of iECoG	
Yes	29 (35.8%)
No	52 (64.2%)
Sex	
Male	41 (50.6%)
Female	40 (49.4%)
Age at surgery	
0–5 years	10 (12.3%)
5–10 years	18 (22.2%)
10–18 years	53 (65.5%)
Mean age at surgery	11.8 years (range 1 to 18 years)
Type of surgery	
Lobectomy	74 (91.3%)
Lesionectomy	7 (87.0%)
Side	
Right	33 (40.7%)
Left	48 (59.3%)

**TABLE 2:** Engel classification by age group and etiology (*n* = 81).

	Engel's classification				% Engel I
	I	II	III	IV	
Age at surgery					
0–5 years (*n* = 10)	8	0	2	0	80.0
5–10 years (*n* = 18)	15	1	1	1	83.3
10–18 years (*n* = 53)	40	7	6	0	75.5
Etiology					
HS (*n* = 34)	25	4	5	0	73.5
FCD (*n* = 22)	17	1	4	0	77.3
Tumor (*n* = 25)	21	3	0	1	84.0

Regarding surgical technique, a standard or tailored anterolateral temporal lobectomy with amygdalohippocampectomy was performed in 74 children (91.3%), with iECoG being used in 24 (29.6%) of these, while the remaining 7 patients underwent lesionectomies (8.7%) cases, guided by iECoG.

**FIGURE 2:**
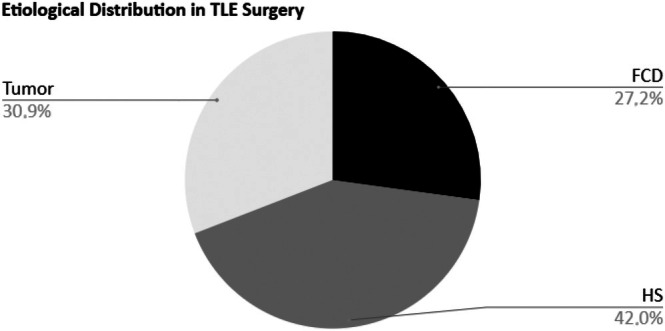
Distribution of different etiologies in pediatric temporal lobe epilepsy surgery.

Thirty‐three (40.7%) operations were performed on the right side; among these, 2 (2.5%) involved focal resections and 31 (38.2%) were lobectomies. In the remaining 48 procedures for left temporal epilepsy, 5 (6.2%) were localized and 43 (53.1%) were temporal lobectomies. Analysis of surgical specimens revealed that HS represented the etiology of epilepsy in a subset of 34 patients (42.0%), while FCD was present in 22 patients (27.2%) and tumors in 25 (30.9%) (Figure 2). The histopathology of tumors was ganglioglioma in 15 (18.5%), dysembryoplastic neuroepithelial tumor (DNET) in 6 (7.4%), low‐grade astrocytomas in 3 (3.7%), and a single patient with FCD and oligodendroglioma.

Overall, among the various etiologies, there were no significant differences in the proportion of Engel I outcomes (*p* = .78) between patients who underwent iECoG (22/29, 75.9%) and those who did not (41/52, 78.8%). For patients with FCD, no differences were seen between those operated with iECoG (10/14–71.4%) or without it (7/8–87.5%) (*p* = .61). In the HS series, 4 of 7 patients (57.1%) monitored with iECoG achieved seizure freedom, as opposed to 21 (77.8%) of 27 patients operated without iECoG who achieved Engel I status (*p* = .35). All patients with tumors who underwent iECoG achieved Engel 1 (8/8, 100.0%), compared to (13/17, 76.5%) who did not use iECoG (*p* = .27) (Table 3).

**TABLE 3:** Engel I outcomes in pediatric temporal lobe epilepsy surgery: Impact of iECoG utilization across different etiologies.

iECoG	Engel's classification				% Engel I	*p* value
	I	II	III	IV		
Total						
Yes	22	1	6	0	75.9	.78
No	41	7	3	1	78.8	
FCD						
Yes	10	1	3	0	71.4	.61
No	7	0	1	0	87.5	
HS						
Yes	4	0	3	0	57.1	.35
No	21	4	2	0	77.8	
Tumor						
Yes	8	0	0	0	100.0	.27
No	13	3	0	1	76.5	

Complications occurred in 6 patients. Of these, 3 cases (3.7%) involved the use of iECoG. There were 2 instances of tonic status epilepticus in the immediate postoperative period and 4 cases of wound infections. Two patients with tumor etiology underwent reoperations. During their initial procedures, they were not monitored with iECoG. However, in their subsequent surgeries, iECoG monitoring was utilized, and both patients achieved Engel IA status.

## 4 DISCUSSION

### 4.1 Surgical effectiveness in pediatric TLE

Resective surgery remains a highly effective treatment for children with drug‐resistant TLE.^1^, ^2^, ^10^ Techniques for intraoperative brain monitoring, such as iECoG, have been proposed to further improve clinical outcomes.^9^, ^11^ In our cohort, seizure freedom rates were up to 80%, regardless of iECoG use. These findings reinforce previous literature data supporting resective surgery as a robust therapeutic option for pharmacoresistant pediatric TLE due to various etiologies.^1^, ^2^, ^12^, ^13^

### 4.2 iECoG in epilepsy surgery

In our center, iECoG is selectively used in cases where noninvasive data are insufficient to define the EZ or when extended resections are anticipated. This approach reflects real‐world practice in many epilepsy surgery centers, where iECoG is used to guide intraoperative decisions in more complex or uncertain cases. Although this brings along an inherent selection bias, it provides a pragmatic framework to evaluate whether iECoG adds clinical value. Our study aimed to assess whether iECoG use, under these real‐world indications, improves seizure outcomes in pediatric TLE. We used high‐density grid electrodes since they seem to provide better coverage of the cortical surface, as reported in recent studies.^14^ The anesthetic protocol was carefully selected, since it is well known that many drugs can interfere with iECoG; for instance, sevoflurane can transiently increase high‐frequency oscillations and spike rates at epileptogenic sites.^15^

The role of iECoG in pediatric epilepsy surgery is not fully established yet, with few studies specifically evaluating its utility in this age group.^16^, ^17^, ^18^ A meta‐analysis by Goel et al., including adult and pediatric cases, found no overall association between use of iECoG and seizure freedom; however, subgroup analysis showed a potential benefit in FCD (hazard ratio = .47; *p* = .037), with no clear advantage in tumor or gliosis‐related epilepsy.^7^

Our study, on the other hand, focused exclusively on pediatric patients, providing new insights about the role of iECoG in this population. In our series—which included patients with FCD, HS, and tumors—Engel I outcomes did not differ significantly between iECoG (75.9%) and non‐iECoG groups (78.8%) (*p* = .78), in keeping with studies questioning the universal prognostic value of iECoG in heterogeneous pathologies.^7^, ^19^, ^20^

Nonetheless, iECoG may offer etiology‐specific benefits. In a cohort of 80 pediatric patients, Lesko et al. found that unresected iECoG abnormalities beyond planned margins in FCD type IIIb were associated with worse outcomes, highlighting the value of a pathology‐specific surgical strategy.^18^

### 4.3 iECoG in temporal lobe tumors

All patients with temporal lobe tumors who underwent iECoG‐guided surgery in our study achieved Engel Ia outcomes, thus increasing seizure freedom rates from 76% (non‐iECoG) to 100% (iECoG), although this was not statistically significant (*p* = .27). This finding is aligned with previous studies suggesting that iECoG may improve outcomes in tumors by allowing for more complete resection of epileptogenic tissue.^21^, ^22^

One hypothesis involves the presence of adjacent FCD type IIIb, which may extend the EZ beyond the visible tumor margins. In such cases, intraoperative neurophysiological mapping becomes crucial for optimizing resection boundaries. Although our study did not include a focused subanalysis on FCD type IIIb, previous studies have demonstrated improved seizure control in pediatric patients with FCD who underwent iECoG‐guided surgery.^17^ Gelinas et al. also reported a seizure freedom rate of 80% within one year of follow‐up in pediatric patients with lesional epilepsy who underwent iECoG‐guided surgery, reinforcing its selective value in structural epilepsies.^20^

Taken together, these data suggest that iECoG may be particularly helpful in tumor‐related epilepsy, especially when subtle or coexisting dysplasia is suspected. Further prospective studies are warranted to confirm its benefit in this context.

### 4.4 iECoG in focal cortical dysplasia

Among patients with FCD Engel I outcomes were comparable between iECoG (71.4%) and non‐iECoG groups (87.5%; *p* = .61). This contrasts with the meta‐analysis by Goel et al., which reported a significant benefit of iECoG in FCD cases (NNT = 4.7; *p* < .001).^7^ However, Goel's study primarily included adult patients, which may account for this discrepancy. Factors such as subtype heterogeneity and immature brain physiology in children likely influence outcomes.

Pediatric epileptogenic networks are often multifocal or diffuse, limiting iECoG's ability to localize deep or dynamic foci. In a meta‐analysis of 1065 pediatric patients, Guo et al. reported 76% seizure freedom with iECoG‐guided surgery, similar to adults (78%; OR = .67; 95% CI: .37–1.20)^23^—highlighting the need for age‐adapted approaches. In addition, modern functional imaging modalities such as fMRI and PET allow accurate localization of EZ, potentially reducing the need for iECoG in selected cases. These data support a selective, etiology‐driven application of iECoG in pediatric epilepsy surgery, particularly in anatomically complex or mixed pathologies.

### 4.5 iECoG in hippocampal sclerosis

Although resection of the mesial temporal structures remains the gold‐standard surgical procedure for cases of TLE, in some patients, mostly in the pediatric age group, the EZ might extend beyond this region. In the present study, subtle FCD could eventually be detected on MRI or metabolic abnormalities could be identified on PET. Nevertheless, in pediatric HS, iECoG showed limited utility, with Engel I outcomes in 57.1% of iECoG cases compared to 77.8% in the non‐iECoG group (*p* = .35). Although not statistically significant, these findings are consistent with the systematic review by Kuruvilla et al. and the results from Schwartz et al., who reported 72% seizure freedom after standard resection despite the frequent presence of extra‐resectional (48%) or residual (38%) spikes.^24^ Notably, neither the presence nor persistence of spikes correlated with surgical outcomes.^19^

These findings, corroborated by those of Goel et al., suggest that iECoG offers no substantial advantage in HS when MRI and EEG are concordant.^7^ In such cases, the EZ is typically well delineated preoperatively, making iECoG dispensable. We recommend restricting iECoG use to MRI‐negative or discordant cases, supporting an etiology‐specific rather than routine approach.

### 4.6 Strengths and limitations

This study benefits from a large single‐center pediatric cohort with long‐term follow‐up, enhancing the reliability of postoperative outcomes. The exclusive focus on pediatric TLE as well as the use of a standardized multidisciplinary evaluation strengthens internal validity. Stratification by etiology enabled a more refined assessment of the potential role of iECoG across different pathological contexts.

However, its retrospective design limits causal inference and introduces potential selection bias. The non‐randomized use of iECoG may reflect confounding by indication. Moreover, small sample sizes within subgroups—particularly in HS—may have reduced statistical power. Finally, the lack of standardization in iECoG methodology may limit reproducibility and generalizability.

## 5 CONCLUSION

iECoG has long been employed to assist epilepsy surgery; however, its role in accurately delineating the EZ remains controversial, particularly in pediatric populations, in whom unique developmental features of epileptogenic substrates may influence outcomes.

In this cohort of 81 pediatric patients undergoing TLE surgery, we found that iECoG did not significantly improve seizure freedom overall. TLE outcomes were achieved across all etiologies, regardless of iECoG use, reinforcing that TLE surgery is a highly effective therapeutic option for pediatric patients with pharmacoresistant epilepsy.

Although iECoG did not demonstrate a clear prognostic benefit in cases of FCD or HS, our findings suggest a potential selective value in tumor‐related epilepsy. This may be related to improved identification of subtle epileptogenic tissue beyond tumor margins, such as coexisting FCD (type IIIb). However, since our study did not include a dedicated subanalysis of dysplasia subtypes and the observed differences were not statistically significant, these findings require cautious interpretation.

These results emphasize the need for a tailored surgical approach based on the underlying pathology and highlight the importance of further prospective studies to better define the specific scenarios in which iECoG may optimize surgical outcomes in pediatric epilepsy.

## AUTHOR CONTRIBUTIONS

Rafael Andrade Cruz (R.A.C.) and João Paulo Sant'Ana Santos de Souza contributed to the study conception, data collection, analysis, and manuscript preparation. Davi Casale Aragon, Úrsula Thomé Costa, Ana Paula Andrade Hamad, and Antônio Carlos dos Santos participated in data acquisition and interpretation. Hélio Rubens Machado and Marcelo Volpon Santos provided overall supervision, conceptual input, and critical revision of the manuscript. All authors read and approved the final version.

## FUNDING INFORMATION

No external funding was received for the completion of this research. However, R.A.C. received a *Scientific Initiation Fellowship* from the São Paulo Research Foundation (FAPESP, Grant No. 2024/07976–8), which provided financial support during the conduction of this study.

## CONFLICT OF INTEREST STATEMENT

The authors declare that the research was conducted in the absence of any commercial or financial relationships that could be construed as a potential conflict of interest.

## PATIENT CONSENT

Written informed consent for surgical procedures and the use of anonymized clinical data for research and publication purposes was obtained from all patients’ legal guardians before study inclusion.

**Test yourself**

1. In pediatric temporal lobe epilepsy, why is defining a clear anatomo–electro–clinical hypothesis critical before surgery?
  - It determines the need for postoperative antiseizure medications
  - It guides the extent and target of surgical resection
  - It predicts neuropsychological recovery after surgery
  - It defines anesthesia management during the procedure
  - It determines the duration of hospitalization
2. From a clinical reasoning perspective, in which situation is intraoperative electrocorticography most likely to add useful information?
  - When MRI and scalp EEG are fully concordant
  - When the epileptogenic zone is well confined to mesial structures
  - When noninvasive data leave uncertainty about cortical involvement
  - When seizure semiology is stereotyped and infrequent
  - When standard temporal lobectomy is planned
3. Why might iECoG have limited impact on seizure outcomes in pediatric hippocampal sclerosis?
  - iECoG cannot detect mesial temporal epileptiform activity
  - Hippocampal sclerosis is usually associated with diffuse epilepsy
  - The epileptogenic zone is often adequately defined preoperatively
  - Pediatric patients have immature cortical electrophysiology
  - iECoG recordings are unreliable under general anesthesia
4. In tumor‐related temporal lobe epilepsy, what is the main pathophysiological rationale for using intraoperative electrocorticography during surgery?
  - To classify tumor histology during resection
  - To identify epileptogenic cortex adjacent to the tumor margins
  - To localize seizure onset zones in remote lobes
  - To confirm complete tumor removal intraoperatively
  - To predict postoperative cognitive outcomes
5. What broader educational principle about surgical adjuncts in epilepsy can be drawn from this study?
  - Intraoperative tools should be applied routinely to all patients
  - More monitoring always leads to better seizure outcomes
  - Surgical success depends mainly on intraoperative findings
  - Adjunctive techniques are most useful when tailored to pathology
  - Long‐term outcomes are independent of presurgical evaluation

*Answers may be found in the* *supporting information*.

## Supporting information


Appendix S1.

## Data Availability

The datasets generated and analyzed during the present study are available from the corresponding author upon reasonable request, in accordance with institutional data‐sharing policies.
