# Supplementary material for: The usefulness of intraoperative electrocorticography (iECoG) in pediatric temporal lobe epilepsy surgery
Source: Epileptic Disord. 2026 Jan 12;28(2):401–8. doi: 10.1002/epd2.70160 (PMC13084204; doi:10.1002/epd2.70160)
Supplement: Supplementary file 1 — Appendix S1. [file EPD2-28-401-s001.docx]

**TEST YOURSELF**

**Answer Key**

Q1 b

Q2 c

Q3 c

Q4 b

Q5 d
